# Does ultrasound provide any added value in breast contouring for radiotherapy after conserving surgery for cancer?

**DOI:** 10.1186/s13014-015-0487-4

**Published:** 2015-08-22

**Authors:** Cynthia Aristei, Isabella Palumbo, Lorenzo Falcinelli, Rossana Crisci, Laura Cardinali, Barbara Palumbo, Valentina Lancellotta, Giampaolo Montesi, Gianni Gobbi, Claudio Zucchetti, Vittorio Bini

**Affiliations:** Radiation Oncology Section, Department of Surgical and Biomedical Science, University of Perugia and Perugia General Hospital, Perugia, 06156 Italy; Radiation Oncology Division, Perugia General Hospital, Perugia, Italy; Radiation Oncology, University of Perugia, Perugia, Italy; Nuclear Medicine, University of Perugia and Perugia General Hospital, Perugia, Italy; Medical Physics Unit, Perugia General Hospital, Perugia, Italy; Internal Medicine, Endocrinal and Metabolic Science, University of Perugia, Perugia, Italy

**Keywords:** Breast conserving surgery, Radiotherapy, Breast contouring, Palpation *vs* ultrasound *vs *CT, Intra-modality variability

## Abstract

**Background:**

Whole breast irradiation after conserving surgery for breast cancer requires precise definition of the target volume. The standard approach uses computed tomography (CT) images. However, since fatty breast and non-breast tissues have similar electronic densities, difficulties in differentiating between them hamper breast volume delineation. To overcome this limitation the breast contour is defined by palpation and then radio-opaque wire is put around it before the CT scan. To optimize assessment of breast margins in the cranial, caudal, medial, lateral and posterior directions, the present study evaluated palpation and CT and determined whether ultrasound (US) provided any added value.

**Methods:**

Twenty consecutive patients were enrolled after they had provided informed consent to participating in this prospective study which was approved by the Regional Public Health Ethics Committee. Palpation and US defined breast margins and each contour was marked and outlined with a fine plastic wire. Breasts were then contoured on axial CT images using the breast window width (WW) and window level (WL) (401 and 750 Hounsfield Units –HU- respectively), at which setting the plastic wires were invisible. Then, the lung window function (WW 1601 HU; WL −300 HU) was inserted to visualize the plastic wires which were used as guidelines to contour the palpable and US breast volumes. As each wire had a different diameter, both volumes were easily defined on CT slices. Results were analyzed using descriptive statistics, percentage overlap and reproducibility measures (agreement and reliability).

**Results:**

*Volumes*: US gave the largest and palpation the smallest. Agreement was best between palpation and CT. Reliability was almost perfect in all correlations. *Extensions:* Cranial and posterior were highest with US and smallest with palpation. Agreement was best between palpation and CT in all extensions except the cranial. Since strong to almost perfect agreement emerged for all comparisons, reliability was high.

**Conclusions:**

US may be useful in defining the cranial and posterior extensions, mainly when tumours are localized there. This study demonstrates that the now standard radio-opaque wires around the palpable breast may not be needed in breast contouring.

## Background

Conserving surgery followed by whole breast irradiation (WBI) is standard local treatment for patients with early-stage invasive, and ductal in situ, breast carcinoma. WBI has evolved over the years, moving from a two-dimensional approach to a three-dimensional (3D) conformal radiotherapy (RT) and advanced techniques, such as intensity modulated RT, volumetric RT, tomotherapy. All modern approaches need target volumes and organs at risk (OAR) of toxicity to be precisely defined [1–7]. The standard approach today uses computed tomography (CT) images so as to minimize the risk of geographic miss and spare OAR such as the lung, contralateral breast and heart in cases of left breast tumours. However, in WBI treatment planning, since fatty breast and non-breast tissues have a similar electronic density, difficulties in differentiating between them hamper volume delineation. Consequently, significant inter- and intra-observer variability was reported [8–12].

One suggestion for overcoming this limitation was to define breast tissue contours by palpation and then put a radio-opaque wire around it before the CT scan [8]. Breast margins, particularly the lateral in obese women, who do not have a clear cleavage plane, are not always easily defined by hand. Atlases using anatomic landmarks [4, 5, 13] were developed but although they reduced inter-observer variability, they could not define breast extension precisely in individual patients [14]. Ultrasound (US) or magnetic resonance imaging (MRI) might define breast margins better than CT or palpation alone [15–17].

In order to optimize assessment of breast margins in candidates for RT after conserving surgery for breast cancer, the present study evaluated palpation and CT and determined whether US provided any added value.

## Methods

Twenty consecutive patients were enrolled after they provided informed consent to participating in this prospective study which was approved by the Regional Public Health Ethics Committee and was conducted in accordance with the Helsinki Declaration of 1975 as revised in 2000.

Median age was 51 years (range 44–75). There were 6 ductal in situ and 14 infiltrating carcinoma (11 T1 and 3 T2; 9 N0, 4 N1 and 1 N2; 6 received adjuvant chemotherapy). Hormonal therapy was prescribed for 15/20 patients.

### Breast margin definition

Breast margins were defined by palpation, US and CT in each patient after immobilization. Patients were supine on a breast board immobilization device, with both arms raised above the head and knee support for comfort.

#### Palpation

Three radiation oncologists, who were experts in breast cancer treatment, performed palpation and reached a consensus agreement on breast margins which were delineated on the skin with a black felt-tip pen.

#### US

A physician who had been trained in ultrasound diagnostics defined the breast margins, using a 2D US scanner (MyLab™Gold 25), and a 12 MHz high resolution probe (LA523) (both from Esaote, Genoa, Italy). After spreading a thin layer of standard US gel (GEL G006 ECO, FIAB, Florence, Italy), the probe was positioned approximately perpendicular to the patient’s skin to define the cranial border and then moved clockwise to define the medial, caudal and posterior margins [17]. During scanning light probe pressure was applied so as not to displace the breast. Breast margins were outlined with a blue felt-tip pen.

#### CT

**Fig. 1:**
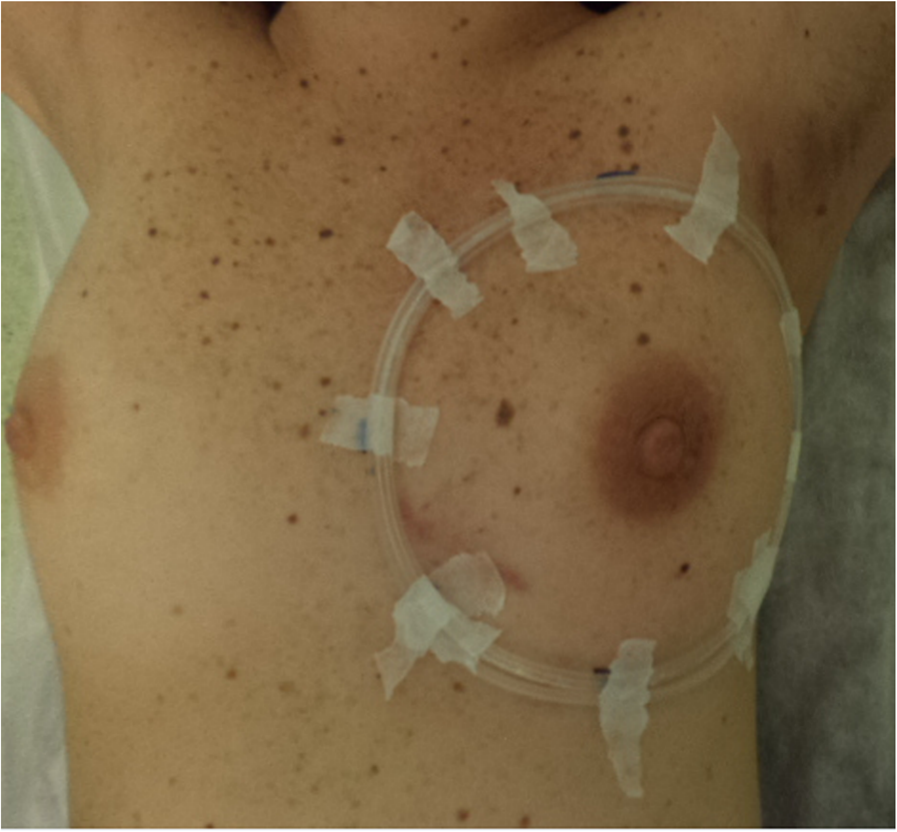
Patient position for palpation, US and CT scans. The figure shows a patient positioned supine on the breast board immobilization device, with two plastic wires in place (the wire used to define palpation margins was 4 mm in diameter, while the wire used to define US margins was 3.33 in diameter)

To avoid artefacts 2 fine plastic wires (4 mm and 3.33 mm in diameter, respectively) were used instead of the standard radio-opaque wires. To ensure palpation and US margins could be distinguished the 4 mm plastic wire was placed on the black palpation outline and the 3.33 mm wire on the blue ultrasound sign. Figure 1 shows a patient as positioned for palpation, US and CT scans, with the two plastic wires in place. A breast scan (Lightspeed QX/I, GE Healthcare) without contrast medium was performed from the mandibular angle or from the lung apex (depending on whether the draining suvra-infra-clavicular nodes had to be irradiated or not) to the diaphragm with 5 mm slice thickness and step. CT-images were then transferred to the treatment planning system (TPS Pinnacle^3^ Philips) for contouring and treatment planning.

Breasts were contoured on axial CT images using the breast window width (WW) and window level (WL) (401 and 750 Hounsfield Units –HU- respectively), at which setting the plastic wires were invisible. WW and WL were changed during delineation when required to improve visualization. The lung window function (WW 1601 HU; WL −300 HU) was inserted to visualize the plastic wires which were used as guidelines to contour breasts according to palpation and US findings. As each wire had a different diameter, both margins were easily defined on CT slices. Reconstructed sagittal and coronal images were used if needed. The three radiation oncologists who had performed palpation agreed on the CT breast margins.

### Breast extension measurement

**Fig. 2:**
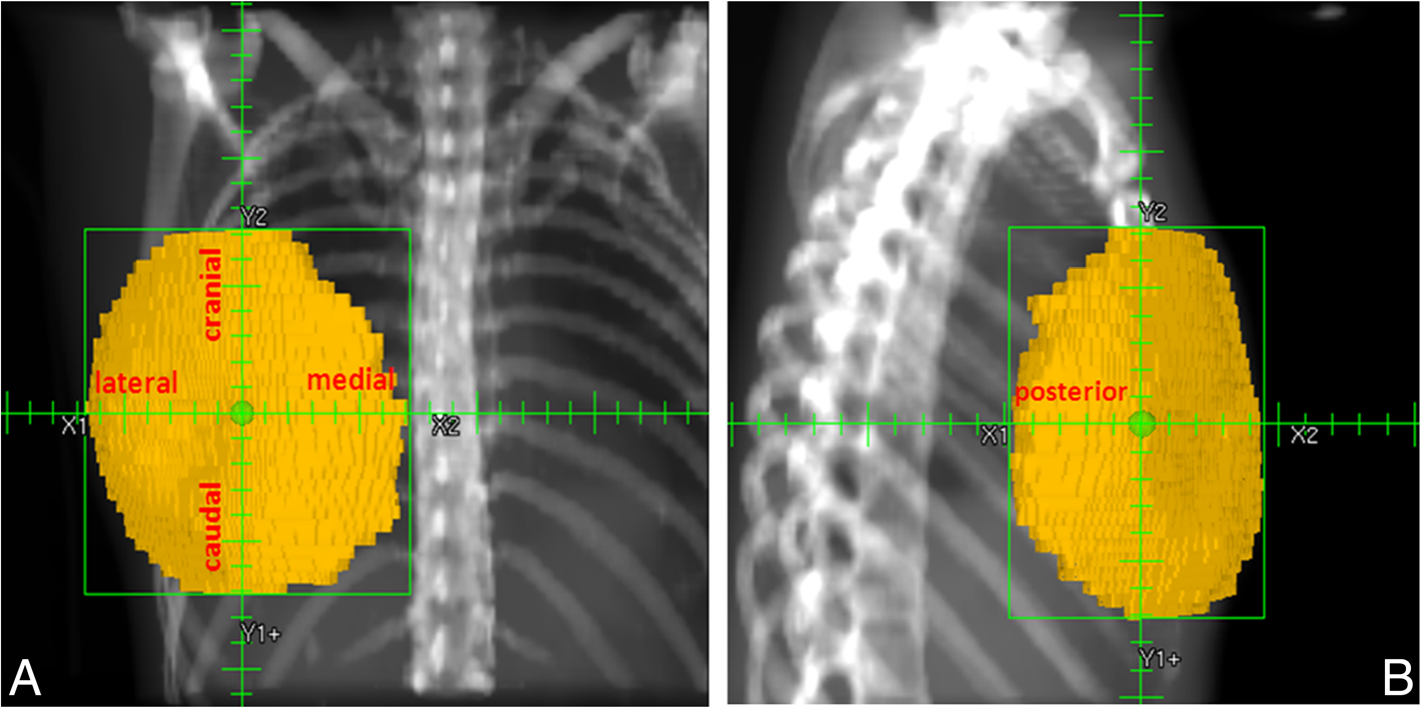
Digitally reconstructed radiography showing: Panel **a** - Anterior field; Panel **b** - Lateral field. The figure shows the point of interest (POI), extensions from the POI (cranial, caudal, lateral, and medial in Panel **a; ** posterior in Panel **b**). Breast volume and the encompassing field are also illustrated. Distances from the POI and the field edges were used to measure breast extension

The most medial, lateral, cranial, caudal and posterior extensions of each breast volume were defined for each patient. A point of interest (POI) was identified on the CT breast volume using a Pinnacle tool (autoplace point option). The placement algorithm uses the smallest possible box to cover the region of interest, i.e. breast volume and then places the POI at the center of this construct. The CT POI was used as a reference point for breast volume as defined by palpation and US. Starting from the POI anterior and lateral orthogonal fields were created to encompass each breast volume. Maximum extensions from the POI in the cranial, caudal, medial and lateral directions were automatically calculated in the anterior field, which was visible on the digitally reconstructed radiography (Fig.2A). Maximum posterior extension was similarly calculated in the lateral field (Fig.2B).

### Statistical analysis

A descriptive analysis was performed, determining mean and standard deviation (SD) of each variable. The percentage overlap (PO) in pairwise comparisons of each volume was calculated, using the formula: PO = V1∩ V2/V1∪V2 [18]. Reproducibility was assessed by means of agreement and reliability measures. Agreement parameters estimate the measurement variability in repeated measurements. Reliability parameters assess whether study objects can be distinguished from each other, despite measurement variability which is related to inter-patient variability.

#### Agreement

Agreement was quantified by calculating the mean difference and SD between US, palpable and CT breast volumes and measurements from the POI to maximum extension in each direction. The 95 % limits of agreement were calculated by the Bland & Altman method [19], with the upper or lower limit of agreement being used to interpret measurement variability. As each modality measurement can be extracted from the other (B-A or A-B), extracting B-A or A-B is an arbitrary decision so + or - signs are irrelevant to result interpretation. Inter-rater differences were plotted against the corresponding mean of two measures for each patient. Kendall’s τ correlation coefficient assessed the interdependence of intra-measure SD and mean.

#### Reliability

The intra-class correlation coefficient (ICC) indicates the inter-patient variance to total variance ratio [20]. It was derived from a random-effects one-way analysis of variance. ICC values are as follow: 0–0.2 indicates poor agreement: 0.3–0.4 indicates fair agreement; 0.5–0.6 indicates moderate agreement; 0.7–0.8 indicates strong agreement; and >0.8 indicates almost perfect agreement [21].

Statistical analyses were performed using IBM-SPSS® version 22.0 (IBM Corp., Armonk, NY, USA, 2013), and StatsDirect version 2.7.2 (StatsDirect Ltd, Altrincham, Cheshire, UK, 2008).

## Results

### Breast volumes

**Fig. 3:**
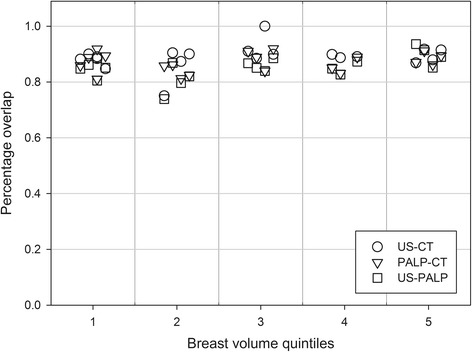
Percentage overlaps in contouring techniques. Percentage overlaps between pairs of contouring techniques (CT *vs* US, US* vs* Palpation, Palpation *vs* CT) according to breast volume quintiles. The percentage overlap variability is independent of breast size

Table 1 shows mean ± SD of breast volumes and their differences as measured by US, palpation and CT. Palpable breast volume was the smallest and the US the largest. POs in pairwise comparisons of each volume were 0.85 ± 0.04 (range 0.74–0.94) for US* vs* palpation, 0.89 ± 0.04 (range 0.75–1) for US *vs* CT and 0.87 ± 0.03 (range 0.81–0.92) for palpation *vs* CT. The scatter plot of POs vs breast volume quintiles shows the PO is independent of breast volume (Fig. 3).

**Table 1:** Breast volumes and extensions as measured by Ultrasound, Palpation and Computed Tomography

		Mean ± SD		Differences in mean ± SD		
	US	PALP	CT	US-PALP	US-CT	PALP-CT
Breast volume (cc)	751 ± 409	674 ± 367	711 ± 370	77.6 ± 66.5	40.5 ± 65.0	−37.1 ± 31.1
Posterior extension (cm)	5.8 ± 1.3	4.8 ± 1.1	5.5 ± 1.1	1.0 ± 0.6	0.3 ± 0.6	0.7 ± 0.5
Medial extension (cm)	8.2 ± 1.4	7.7 ± 1.4	8.1 ± 1.3	0.5 ± 0.5	0.1 ± 0.4	−0.4 ± 0.4
Lateral extension (cm)	7.8 ± 1.4	8.1 ± 1.3	7.8 ± 1.2	0.2 ± 0.3	0.1 ± 0.3	0.0 ± 0.2
Cranial extension (cm)	9.5 ± 1.2	8.7 ± 1.2	9.0 ± 1.0	0.8 ± 0.6	0.5 ± 0.5	−0.3 ± 0.6
Caudal extension (cm)	9.6 ± 1.1	9.0 ± 1.1	8.9 ± 1.0	0.5 ± 0.4	0.7 ± 0.6	0.2 ± 0.4

Abbreviations: *US* Ultrasound, *PALP* Palpation, *CT* Computed tomography

**Fig. 4:**
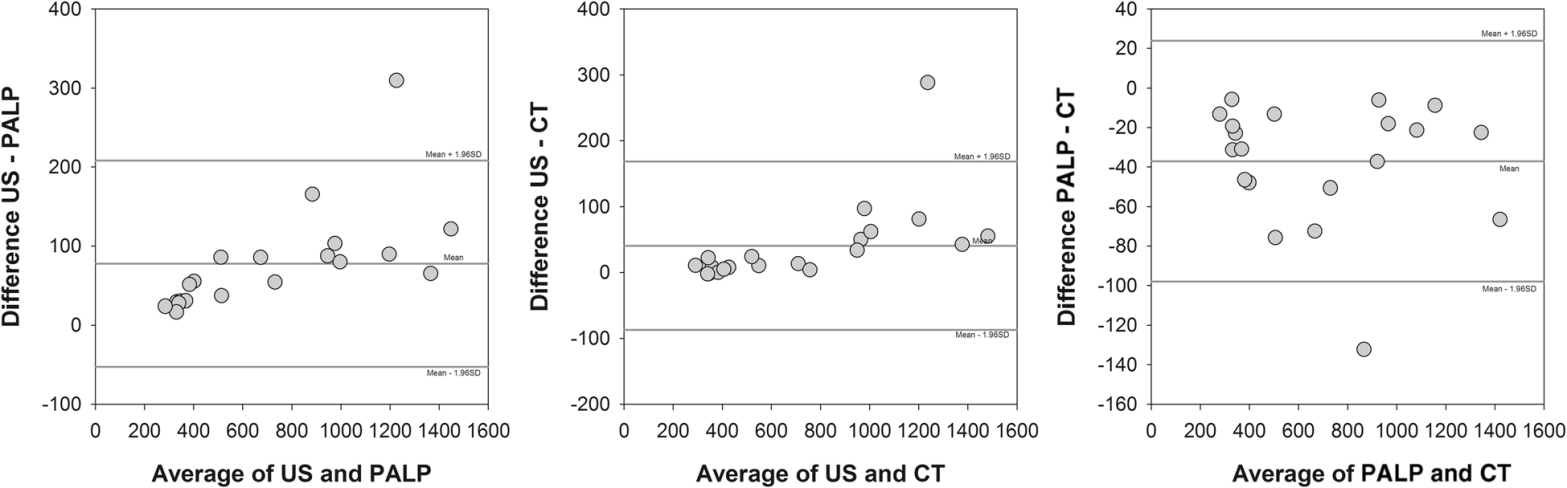
Bland-Altman agreement plots. Breast volume differences were plotted against the mean breast volume for each pair of contouring techniques, with each point representing one patient. The horizontal lines indicate the mean difference (middle line) and the 95 % limits of agreement. The smaller the range between these two limits the better agreement is

**Fig. 5:**
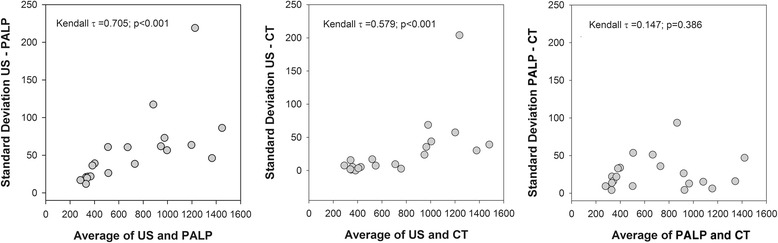
Kendall’s τ correlation coefficients. Kendall’s τ correlation coefficient established whether measurement variability depended on measurement size assessing the interdependence of intra-measure mean and standard deviation (SD)

Table 2 reports the results of inter-rater agreement, Kendall’s τ correlation coefficient and reliability, showing almost perfect agreement in ICC for all modality pairwise comparisons. Figure 4 illustrates inter-rater agreement when differences in breast volumes were plotted against their mean value (each point represents one patient). There was only 1 outlier. Figure 5 shows Kendall’s τ correlation coefficient between breast volume means and SD in pairwise comparisons was significant in US *vs* palpable and US *vs* CT (*p* < 0.001 for both correlations). Palpable *vs* CT were not significantly correlated (*p* = 0.386).

**Table 2:** Ultrasound, Palpation and Computed Tomography: Inter-rater agreement and reliability

	Lower and upper limits of agreement			Kendall’s τ			ICC		
	US-PALP	US-CT	PALP-CT	US-PALP	US-CT	PALP-CT	US-PALP	US-CT	PALP-CT
Breast volume (cc)	−52 to 208	−87 to 168	−98 to 23	0.705^*^	0.579^*^	0.147	0.965	0.981	0.991
Posterior extension (cm)	−0.27 to 2.20	−0.88 to 1.38	−1.65 to 0.22	0.275	0.222	0.026	0.605	0.869	0.732
Medial extension (cm)	−0.38 to 1.44	−0.64 to 0.93	−1.20 to 0.42	−0.064	0.369°	−0.251	0.880	0.950	0.913
Lateral extension (cm)	−0.52 to 0.83	−0.44 to 0.70	−0.43 to 0.38	0.018	0.249	0.032	0.950	0.963	0.983
Cranial extension (cm)	−0.44 to 2.04	−0.42 to 1.40	−1.40 to 0.78	0.091	0.289	−0.395	0.666	0.827	0.834
Caudal extension (cm)	−0.35 to 1.41	−0.48 to 1.91	−0.57 to 0.95	0.032	−0.150	−1.185	0.810	0.661	0.918

Abbreviations: *US* Ultrasound, *PALP* Palpation, *CT* Computed tomography, *ICC* Intraclass correlation coefficient

^*^ *p* < 0.001; °*p* < 0.05

### Breast extensions

Extension results are reported in Tables 1 and 2. Briefly, palpation measured the smallest and US the highest mean cranial and posterior extensions, with results from CT in the middle. Inter-rater agreement was better when palpation and CT were compared, except for the cranial extension, where major concordance was observed between US and CT. In the medial extension, there was good agreement between all modalities. Kendall’s τ correlation coefficient showed only mean and SD of medial extension on US and CT correlated significantly (*p* < 0.05). ICC-values ranged from 0.605 to 0.983, showing that agreement was from strong to almost perfect in all correlations between the different modalities.

## Discussion

The present investigation assessed palpation and CT in breast contouring and, by evaluating inter-modality variability, determined whether US provided any added value in the postoperative RT work-up.

US was evaluated *vs* anatomic references and palpation as a tool for defining breast margins in the 2D era [17], assuming US volumes as real. Unlike the present study, CT was not used, analyses were qualitative and measurements were not compared. Objections may be made to present use of 5 mm CT slice thickness, rather than 2 mm as the most recent guidelines suggest [6], on the grounds that it could influence cranial and caudal margin estimates. Since the 5 mm CT slice thickness was being used in our institute when we designed this study protocol we continued with it. Furthermore with this thickness, both the 4 and 3.33 mm plastic wires were visualized on one slice instead of being split over two, thus eliminating a potential confounding variable.

In our study contouring results showed that US defined the greatest mean volume and palpation the smallest, maybe because it is more difficult to distinguish breast volume from non-breast fat with palpation. On the other hand echogenicity varies with breast tissue, fibro-adipose tissue, muscles and the subcutaneous layer, so US can easily distinguish each different type of tissue and accurately identify breast extensions.

Despite the difference in breast volumes, all percentage overlaps were large, showing little differences in estimates by any technique. To strengthen objectivity, reproducibility was assessed by means of agreement and reliability. Both analyses had been used mainly to evaluate inter- and intra- observer variability in contouring target volumes or organs at risk of toxicity [22, 23].

Agreement parameters, which are linked solely to the instrument, showed how close the results of the repeated measurements were and the inter-rater agreement indicated that, although US volumes differed most from the other two, all values were confined within the 95 % CI with only one outlier. Consequently, variations from the mean in each pairwise comparison depended on modalities. Kendall’s τ correlation coefficient, which illustrated the link between mean and SD in repeated measurements, indicated that the greatest variability was found with US because it provided higher mean volumes than the other modalities. Reliability parameters, which are highly dependent on study sample heterogeneity, determined whether volumes could be distinguished from each other. Consequently, in the present study, reliability measured how classification of breast volumes from largest to smallest or *vice versa* as determined by one modality correlated with another. Reliability was high, as the ICC indicated almost perfect agreement for all correlations. Thus the difference in US results had no appreciable impact upon classification.

As far as regards extensions, all modalities yielded similar measurements in the five spatial directions except for the cranial and posterior. US gave the highest values and palpation the lowest with CT in the middle, accounting for the pattern in volume measurements. Inter-rater agreement was strong in the medial extension when all modalities were tested in pairwise comparisons. It was best for palpation *vs* CT in all extensions but the cranial. As observed for volumes, reliability was high, as ICC showed strong to almost perfect agreement for all evaluations. Thus the evidence suggests that cranial and posterior extensions are critical because there it is more difficult to differentiate between breast and non-breast tissues. In fact previous studies showed inter-observer variability was highest in these areas when breast tissue was contoured on CT images [8–11, 24]. To reduce variability in the contouring process Hurkmans et al. first placed the now standard radio-opaque wires on the skin around palpable breast [8]. Even though they are considered extremely helpful in defining breast margins on CT scans, our results suggest they are probably not really necessary as we found high agreement between palpation and CT. If confirmed in a larger cohort of patients this finding may lead to changes in guideline recommendations for breast contouring.

To improve breast contouring when margins are hard-to-define, atlases were developed using anatomical landmarks [4, 5, 13]. Although helpful, they are surrogate markers of breast margins which, however, have great inter-individual differences that are linked to age, menopausal state, parity, weight, and so on. Consequently, breast contouring by landmarks may risk volume over- or under-estimation.

Imaging modalities which define breast margins better might overcome inter-observer variability in contouring. Even though MRI is now standard for some organs, few studies have evaluated it in breast contouring. As we found with US, volumes defined with MRI shifted mainly in the cranial or posterior directions compared with CT [15, 16]. Furthermore, expensive, time-consuming MRI is not easy to use in treatment planning: not all radiation oncology centres have dedicated MRI; images must be co-registered with CT so the patient must be in the treatment position on a flat bed with all necessary devices [25]. Because of these limitations, the present study assessed the US contribution to contouring. Advantages included scanner location in the simulation room; US was quick, cheap and easy to use; the patient could be scanned immediately before the CT scan on the same bed using the same immobilization device as for CT and therapy. In any case, changes in patient position between the two scans have to be considered and attention has to focus on patient set-up in accordance with standard protocols.

Despite these practical advantages, no evidence emerged to support routine use of US in breast contouring. Percentage overlap between volumes defined by US, palpation and CT was large and agreement ranged from strong to almost perfect. In our view US might be most suitable for selected patients whose tumours were originally located in the superior and external parts of the breast. Indeed US gave the highest values in these difficult to define cranial and posterior extensions, as echogenicity clearly varied with the tissues.

## Conclusions

To sum up, the practical applications of this study is that radio-opaque wires around palpable breast may not be needed in breast contouring for treatment planning in RT candidates. Furthermore, even though a clear role did not emerge for US, it may usefully supplement CT in defining the cranial and posterior extensions, particularly when tumours were originally localized there.

## Abbreviations

WBI: Whole breast irradiation
3D: Three-dimensional
RT: Radiotherapy
OAR: Organs at risk
CT: Computed tomography
US: Ultrasound
MRI: Magnetic resonance imaging
TPS: Treatment planning system
WW: Window width
WL: Window level
HU: Hounsfield Units
POI: Point of interest
SD: Standard deviation
PO: Percentage overlap
ICC: Intra-class correlation coefficient
